# Vernal Keratoconjunctivitis among Patients Presenting to the Outpatient Department of Ophthalmology of a Tertiary Care Centre: A Descriptive Cross-sectional Study

**DOI:** 10.31729/jnma.7933

**Published:** 2023-01-31

**Authors:** Neha Priyadarshani Chaudhary, Badri Prasad Badhu, Prabhat Deo

**Affiliations:** 1 Department of Ophthalmology, Birat Medical College Teaching Hospital, Tankisinwari, Biratnagar, Nepal

**Keywords:** *conjunctivitis*, *refractive error*, *vernal keratoconjunctivitis*

## Abstract

**Introduction:**

Vernal keratoconjunctivitis is a seasonally recurring, bilateral inflammation of the conjunctiva, that occurs in male children with invariable personal or family history of atopy. It is characterized by interstitial inflammation of the cornea and can have sight-threatening complications if not treated in time. The aim of this study was to find out the prevalence of vernal keratoconjunctivitis among patients presenting to the outpatient department of ophthalmology of a tertiary care centre.

**Methods:**

This descriptive cross-sectional study was conducted among patients presenting to the outpatient department of ophthalmology from June 2020 to May 2021. Ethical approval was taken from the Institutional Review Committee (Reference number: IRC-PA-076). The relevant details of the history and clinical examination of the patients were recorded on a specifically designed proforma. A simple random sampling technique was used. Point estimate and 95% Confidence Interval were calculated.

**Results:**

Among2400patients with conjunctivitis visiting the outpatient department of ophthalmology, vernal keratoconjunctivitis was seen in 80 (3.33%) (2.61-4.05, 95% Confidence Interval).

**Conclusions:**

The prevalence of vernal keratoconjunctivitis in our study was found to be similar to the other studies done in similar settings.

## INTRODUCTION

Vernal keratoconjunctivitis (VKC) is a bilateral, recurrent inflammation of the conjunctiva that tends to occur in children and young adults. Its onset is most common in the spring and goes into remission during the cooler months.^1^ The highest incidence of the disease is in the warm, temperate Middle East Mediterranean region and Mexico. Boys are affected twice as often as girls with a peak incidence between the ages of 11 and 13 years. Typical symptoms are ocular itching, watering, foreign body sensation and mucoid discharge.^2^

The three forms of vernal conjunctivitis are palpebral, limbal, and mixed.^3^ Corneal manifestations include a superficial pannus and punctate epithelial keratitis. In severe cases, the cornea appears to be dusted with flour. The long-term prognosis is generally good; however 6% of patients develop corneal damage, cataract, or glaucoma.^4^ As untreated VKC can lead to permanent visual loss, we should be aware of allergens and the management and therapeutic options for this disease to allow patients to enter clinical remission with the least side effects and sequelae.

The aim of this study was to find out the prevalence of vernal keratoconjunctivitis among patients presenting to the outpatient department of ophthalmology of a tertiary care centre.

## METHODS

This descriptive cross-sectional study was conducted among patients presenting to the outpatient department of ophthalmology of Birat Medical College Teaching Hospital from June 2020 to May 2021 after obtaining ethical approval from the Institutional Review Committee (Reference number: IRC - PA-076). Informed verbal consent was taken from all the cases in adult patients and from parents in cases of children before taking the cases for the study. All patients with conjunctivitis were included in the study. The patients having any other ocular pathology other than conjunctivitis, patients more than 18 years of age and patients with unilateral involvement were excluded. A simple random sampling technique was used. The sample size was calculated using the following formula:


$$\begin{array}{ll} \text{n} & ={\text{Z}}^{2}\times \frac{\text{p}\times \text{q}}{{\text{e}}^{\text{2}}}\\ & =1.96^{2}\times \frac{0.50\times 0.50}{0.03^{2}}\\ & =2401 \end{array}$$

Where,

n = minimum required sample size
Z = 1.96 at 95% Confidence Interval (CI)
p = prevalence taken as 50% for maximum sample size calculation
q = 1-p
e = margin of error, 3%

The minimum sample size obtained was 1068. However, the final sample size taken was 2400.

The patient's demographic information included date of presentation to the hospital, address, age, sex, occupation, presenting complaints, month of presentation, duration of disease, age of onset, previous drug administration and any other allergic disorder. A pen torch and slit lamp biomicroscope were used for assessing the anterior segment while direct and indirect ophthalmoscopes were used for posterior segment evaluation. The diagnosis of VKC was made based on the basis of the history of itching and burning, redness or brownness, lacrimation, photophobia and a mucinous, ropy discharge and or clinical presence of papillae in the lower or upper tarsal conjunctiva, limbal papillae and trantas spots. The vision and baseline refractive status of all children was recorded accordingly.

Data collected were entered and analyzed using IBM SPSS Statistics version 25. Point estimate and 95% CI were calculated.

## RESULTS

**Figure 1:**
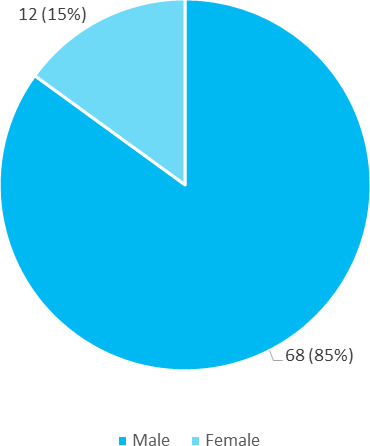
Gender distribution of patients with vernal keratoconjunctivitis (n= 80).

Among 2400 patients of conjunctivitis attending the outpatient Department of Ophthalmology, vernal keratoconjunctivitis was seen in 80 patients (3.33%) (2.61-4.05, 95% CI). Out of 80 patients, 68 (85%) were male and 12 (15%) were female (Figure 1). The male: female ratio was 5.6:1.

Majority of patients, i.e. 45 (56.25%) were in the 7-12 years of age, 32 (40%) were in the age group of 0-6 years and 3 (3.75%) of patients were in the age group of 3-18 years.

**Figure 2:**
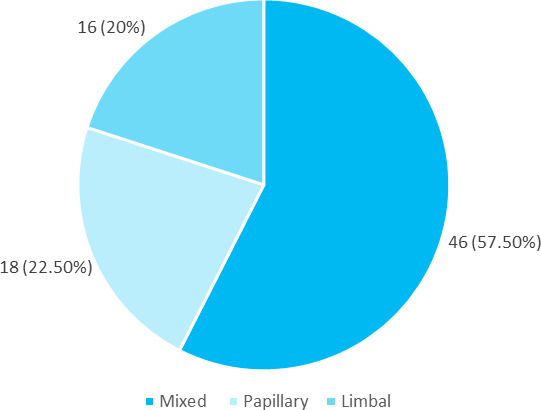
Different types of VKC observed (n= 80)

The most common type of VKC was found to be mixed type which was seen in 46 (57.50%) patients, followed by limbal which was seen in 18 (22.50 %) and papillary type was seen in 16 (20%) (Figure 2).

The most common presenting symptom was itching which was seen in 79 (98.75%) patients followed by redness which was seen in 52 (65.00%) patients. The most common sign seen in these patients was the presence of conjunctival hyperemia 56 (57.50%) (Table 1).

**Table 1:** Symptoms and signs among patients with VKC (n= 80).

Symptoms	n (%)
Itching	79 (98.75)
Redness	52 (65.00)
Watering	28 (35.00)
Discharge	15 (18.75)
**Signs**	
Conjunctival hyperemia	56 (57.50)
Tarsal papillae	52 (65.00)
Limbal papillae	52 (65.00)
Conjunctival pigmentation	41 (51.25)
Trantas spot	17 (21.25)
Shield ulcer	3 (3.75)
Filamentary keratitis	1 (1.25)
Pseudoxerontoxon	7 (8.75)

In 70 patients (87.50%), the disease showed a seasonal predominance with clinical presentations seen mainly in summer seasons whereas in the remaining 10 patients (12.50%), it was perennial in nature (Table 2).

**Table 2:** Seasonal variation (n= 80).

Onset	n (%)
Seasonal	70 (87.50)
Perennial	10 (12.50)

Out of 80 patients, 74 (92.5%) were above 4 years of age who were subjected to Snellen's visual acuity charts for assessingtheir vision. In the remaining 6(7.5%) children, theirvision was assessed by looking attheir ability to follow and fixate light. The visual acuityof 74 (92.5%) childrenin both the right eye and left eyewas recorded (Table 3).

**Table 3:** Visual acuity in the right eye and left eye (n= 74).

Visual acuity	n (%)
**Right eye**	
6/6-6/12	63 (85.13)
<6/12-6/18	6 (8.10)
<6/18-6/60	3 (4.05)
<6/60-3/60	2 (2.70)
**Left eye**	
6/6-6/12	66 (89.20)
<6/12-6/18	4 (5.40)
<6/18-6/60	3 (4.10)
<6/60-3/60	1 (1.40)

## DISCUSSION

Our study showed that VKC in this part of the country is essentially similar to the demographic profile and clinical presentation described in other tropical countries.^5^

The prevalence of vernal keratoconjunctivitis in our study was 3.33% which is similar to other studies in other countries in the African continent like Rwanda (Central Africa) in the year 2011, where the prevalence was 4%,^2^ and in Ethiopia (2012), where it was found in 5.2% of children between 11 and 15 years.^6^

Majority of patients, i.e. 45 (56.25%) were in the 7-12 years of age, 32 (40%) were in the age group of 0-6 years and 3 (3.75%) of patients were in the age group of 3-18 years. In a study done in India, persistent disease beyond 20 years of age was reported in 12% of patients and the mean age of presentation in their study was 12 years.^7^ The gender distribution showed that 68 (85%) of the patients were males and the remaining 12 (15%) were females. The male: female ratio was 5.6:1 in this study. In a study done among the Italian population, the M: F ratio was found to be 3.5:1.^8^ Similarly, another study reported M:F ratio of 3.3:1 in a demographic and epidemiological study involving a case series of 406 VKC patients.^9^ This variation may be because of the difference in sample size, racial variation, and geographical distribution.

The majority of patients had a VKC of the mixed type (58%), the limbal form of the disease accounted for 22%, while the palpebral form made up 20% of the total number of patients. The multi-centric study from Italy reported predominance (53.8%) of limbal presentation, whereas study done in India, found the majority of the cases (71.8%) had a mixed presentation comprising of both limbal as well as palpebral involvement, followed by isolated palpebral involvement in 15.6% and limbal involvement in 12.6% of the patients.^7,8^

Itching was the commonest symptom seen in 79 (98.75%) patients followed by redness 52 (65%), watering 28 (35%) and discharge 15 (18.75%). In another study also almost similar results, itching (88%), redness (86%) and watering (65%) were reported.^10^ Similar results were also reported by another study done among school children in Rwanda.^2^

Majority of patients, i.e. 56 (70%) had Conjunctival hyperemia, 52 (65%) had papillae on the upper tarsal conjunctiva and gelatinous limbal thickening with papillae was seen in 52 (65%) patients. Another study done in India found tarsal papillae in 85% of patients and limbal thickening in 73% of patients with VKC.^5^ Perilimbal conjunctival pigmentation is a new clinical sign described in VKC.^11-13^

In all forms of allergic eye diseases, the clinical response is caused by mast-cell activation due to either an antigen-mast cell linkage or T-cell activation of mast cells. The activation of conjunctival mast cells leads to the release of histamine, prostaglandin D2, leukotriene C4, tryptase, chymase, platelet-activating factor, and other chemo-attractants. This further attracts eosinophils and neutrophils.^14-16^

The limitations of this study could be the lack of follow-up of the patients since the study design was a descriptive cross-sectional study. Also, it would be better to have a multicentric study for better precision. VKC with atypical presentation and clinical features might have been misdiagnosed.

## CONCLUSIONS

The prevalence and the clinical presentation of VKC patients was found to be similar to other studies done in similar settings. As untreated VKC can lead to permanent visual loss, the clinical presentation, its pathogenesis and treatment modalities are very important to prevent blindness.
